# How effective are interventions in optimizing workplace mental health and well-being? A scoping review of reviews and evidence map

**DOI:** 10.5271/sjweh.4087

**Published:** 2023-05-01

**Authors:** Alex Waddell, Breanne Kunstler, Alyse Lennox, Loyal Pattuwage, Emily AC Grundy, Diki Tsering, Patrick Olivier, Peter Bragge

**Affiliations:** 1 Monash Sustainable Development Institute Evidence Review Service, Monash University, Australia.; 2 BehaviourWorks Australia, Monash Sustainable Development Institute, Monash University, Australia.; 3 Department of Human-Centred Computing, Faculty of Information Technology, Monash University, Australia.

**Keywords:** burnout, practice review, stress

## Abstract

**Objectives:**

Mental well-being is critical to quality of life. Workplace mental well-being is crucial to ensure employee health, satisfaction, and performance. Mental ill-health is a global challenge, costing workplaces $17 billion per year. Workplaces have realized the need for investment in interventions to promote mental health and well-being in their workforce. However, given their limited resources, workplace personnel responsible for program implementation need evidence-based guidance on which interventions influence which outcomes.

**Methods:**

This study employed a scoping review methodology in order to produce an evidence map and includes reviews of workplace mental well-being interventions. The search strategy focused on peer-reviewed articles with the primary aim of investigating workplace mental health interventions. Reviews were assessed for quality using AMSTAR 2. The evidence map includes interventions (rows) and outcomes (columns), with the relative size of the reviews underpinning each intersection represented by circles and the direction of evidence represented by color.

**Results:**

Eighty reviews were deemed eligible from 4795 citations. The resulting evidence map includes 17 intervention types designed to influence 12 outcomes. Interventions with the highest quality evidence were mindfulness, education and information provision, and individual psychological therapies. The most common outcomes were burnout / stress reduction and mental well-being. Interventions tended to focus on individual level factors rather than organizational or system-level factors.

**Conclusion:**

The evidence-base for workplace mental health interventions is broad and extensive. There is an apparent knowledge-to-practice gap, presenting challenges to implementing workplace mental health programs (ie, what interventions have the highest quality evidence). This study aims to fill the gap by providing an interactive evidence-map. Future research should look to fill the gaps within the map including the lack of organization and system level factors and especially economic evaluations.

Mental Health is defined by the World Health Organisation (WHO) as *“a state of well-being in which an individual realizes his or her own abilities, can cope with the normal stresses of life, can work productively and is able to make a contribution to his or her community”* (1). Importantly, the WHO also emphasize that mental health is more than the absence of disorder. Therefore, terms such as ‘mental well-being’ and ‘thriving’ are often used interchangeably with the term ‘mental health’ (2–5).

Workplace mental health and well-being is a broad concept focused primarily on the health of the workforce, which can consequently determine good or poor outcomes in the workplace. Having high workplace mental health and well-being is important to ensure optimal health of employees and can contribute to high employee satisfaction and performance, and employees dedicated to achieving the goals of their organization (6). Conversely, lower workplace mental health and well-being contributes to lower employee satisfaction and performance, and may lead to burnout, lower mental well-being and increased mental illness (6). *Burnout* is an occupational phenomenon, rather than a clinical condition, in which chronic workplace stress is not appropriately managed and leads to feelings of exhaustion, feelings of apathy or negative affect toward one’s job, and reduced productivity (1). Mental well-being or mental health problems are characterized by changes to a person’s ability to think, feel and behave and do not constitute mental illness, but may lead to mental illness. Mental illnesses are diagnosable conditions that significantly affect the functioning of a person and how they think, feel and behave (7).

Mental ill-health is a global challenge, contributing substantially to the global burden of disease (8). Approximately 2.8 million working Australians experience mental ill-health (9). These challenges can impair an individual’s ability to attend work or to function effectively when they do attend (10, 11), costing workplaces an estimated $17 billion (Australian dollars) a year (9). While employment can cause or exacerbate mental ill-health (12), workplaces also have the opportunity to help prevent or mitigate it (13, 14). The Australian Productivity Commission is calling for reforms to “equip workplaces to be mentally healthy” (9). Yet, despite growing recognition of the importance of workplace initiatives, there is insufficient guidance on what works to promote mental health in these settings (15).

Many workplaces have realized the need for investment in workplace mental health programs, especially given the positive return on investment, which is estimated to be a $2.3 return for every $1 spent in Australia (16). Connectedness, culture, capability, leadership and policies that focus on enhancing workplace mental health and well-being and address psychosocial risks (eg, inappropriate workload) that can determine poor outcomes can all contribute to the well-being of a workplace (17). A survey of 10 009 Australian workers reported that large organizations (1000–4999 employees) experienced a decline in mental health and well-being from 2020 to 2021 compared to 'huge' workplaces (≥5000 employees) in which mental health and well-being remained steady (12). Policy and capability gains were reported to protect huge workplaces from lower mental health and well-being. The declines have been attributed to leadership and connectedness decreasing with remote work and the limited visibility of the implementation of mental health policies, making discussing mental health concerns in the workplace more challenging (17). Thus, it is important to identify effective ways to improve mental health and well-being in these settings by identifying effective interventions and to support those charged in delivering them.

However, considering that knowledge takes many years to become practice (known as the knowledge-to-practice gap) (18) many organizations require guidance on which interventions they should implement to improve mental health and well-being in the workplace. Workplaces have limited resources to invest in addressing this problem, it is important to determine which interventions are most effective based on the existing research. That is, which interventions influence what outcomes and to what degree (14).

In Australia, workplace managers and human resources (HR) staff are responsible for designing and implementing policies intended to enhance and maintain the mental health and well-being of employees. This suggests that management and HR staff could benefit most from the identification of evidence-based interventions to support workplace mental health and well-being. As such, the aims of this scoping review and evidence map were to: (i) identify, appraise and synthesize research evidence exploring the efficacy of workplace mental health and well-being interventions across a broad spectrum of intervention categories, settings and outcomes; and (ii) display the available research in an interactive and accessible manner for ease of use by practitioners, researchers and other stakeholders.

In this paper, we use the term ‘mental well-being’ as an all-encompassing reference to the WHO and other definitions outlined above.

## Methods

### Evidence mapping methods

Given the breadth of workplace mental well-being challenges and interventions that may address them, access to an overview of the research field by employees, employers, policymakers and other groups may be helpful. Traditional systematic reviews are not well-suited to this aim because they are designed to explore specific topics in depth (19). For example, a systematic review may aim to establish effectiveness of cognitive behavioral therapy for addressing acute anxiety by finding, appraising and synthesizing all primary studies that have undertaken this evaluation.

Evidence mapping, which is based on established methods of scoping reviews (20), is a preferable approach. Evidence mapping aims to provide an overview of an entire research field (21, 22). Results of evidence mapping are often presented as visual ‘evidence maps’ representing the research landscape – for example with rows representing interventions, columns representing outcomes and each cell quantifying and characterizing the research relating to each intervention-outcome pair. In our evidence map, each cell’s color is determined by the direction of the evidence from the reviews referenced within that cell. The conclusions of reviews existing within the same cell were considered together to determine the final color of that cell. Review conclusions were either gained through narrative or statistical analysis and were either neutral (no change in outcome = grey), negative (opposite of the desired effect of the intervention = red), positive (intervention had a desirable effect on the outcome = green) or no conclusion (= orange).

These visual evidence maps allow users to quickly digest large amounts of information about the evidence available. Evidence maps carry the dual advantage of illustrating areas in which evidence-based action can be taken as well as where future research is required (23).

The unit of analysis for the evidence map was reviews of workplace mental well-being interventions. A ‘review of reviews’ approach is highly compatible with evidence mapping given the size of the workplace mental well-being field. This method enables literature on a broad array of interventions and health outcomes to be synthesized. Furthermore, overviews of reviews are designed for decision-making audiences including providers, policymakers and informed consumers (24–26).

The evidence mapping approach and underlying review employed best-practice techniques and reporting standards (27). A protocol outlining key review parameters was registered on the Open Science Framework in October 2020 (https://osf.io/ymzvh). In summary:

*Question.* An explicit review question was finalized: What is the effectiveness of workplace mental health interventions?

*Search.* A comprehensive search of Ovid Medline and APA PsycInfo from 1 January 2016 to 12 October 2020 and Google Scholar (first 100 citations, sorted by relevance) was undertaken (see supplementary material, URL).

*Selection*. Two authors independently conducted a title / abstract and full text screening against pre-determined inclusion / exclusion criteria. Articles were included if they were systematic reviews, meta-analyses, systematic reviews and meta-analysis, or scoping reviews of workplace mental well-being interventions conducted in countries comparable to Australia since 2016. Countries comparable to Australia exclude those that are non-democratic and/or low- and middle-income countries as defined by the World Bank (28). Reviews that measured changes in outcomes specific to mental health were included (eg, wellbeing) and, provided mental health parameters were measured as outcomes, the interventions could have any component (for full criteria see supplementary material). Across all screening, any discrepancies were discussed until consensus was reached or resolved by a third reviewer

### Quality appraisal

Quality appraisal was undertaken using AMSTAR 2 (29). Two authors independently assessed the quality of included reviews. Reviews scoring ≥50% were deemed high quality. Any discrepancies were discussed until consensus was reached or resolved by a third reviewer.

*Data extraction.* Two authors independently extracted data from the included articles. Data extracted included citation; study (review) type; professional group; direction of evidence; interventions; and outcomes studied.

Interventions studied in the included reviews were iteratively coded according to their descriptions in the reviews under the following broad categories (ie, intervention types were added as they appeared using self-described language used by the review authors): mindfulness / meditation (including relaxation; diet; sleep; exercise; psychological therapies (individual); psychological therapies (group); peer support; pharmacotherapies; physical environmental modifications; organizational modifications; education / information provision; team-building activities; spiritual / religious; arts-based interventions (including music); complementary and alternative therapies (eg, aromatherapy); virtual reality; not described; and mixed (eg, digital health interventions involving multiple components).

A similar approach was used for outcomes: mental well-being; mental illness; absenteeism; burnout / stress reduction; productivity; employee satisfaction; economic outcomes; life purpose and satisfaction; physical health; attitudinal and/or emotional measures; and fatigue.

Occupations were defined using the Australian and New Zealand Standard Industrial Classification (ANZSIC) 2006 [revision 2.0, 2013 (30)].

*Evidence mapping.* The evidence map was designed to enable users to interactively access information across multiple interventions and outcomes. The Tableau data visualization tool was used to render the map (31). Navigation within the evidence map was based on Rayasam et al (32).

## Results

### Study selection

**Figure 1:**
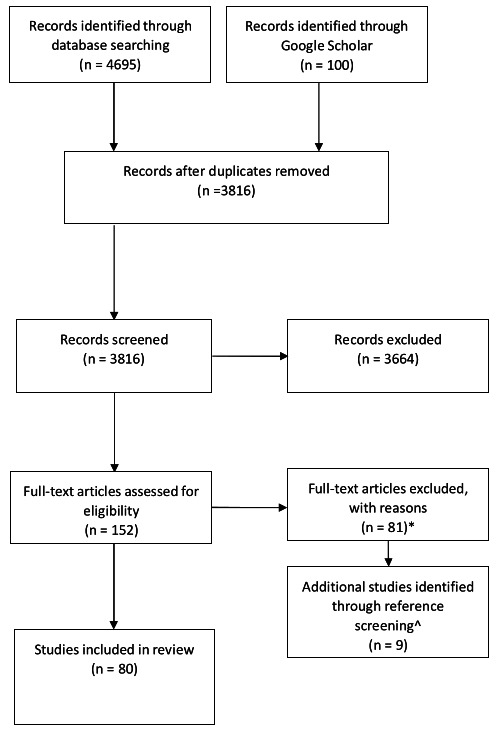
PRISMA flow diagram. *Of the 152 articles reviewed, N=54 were excluded as the wrong study type, N=12 were exclude as they did not include studies of employees, N=10 were excluded as they were the incorrect intervention type, N=5 were excluded as they did not include the outcome of interest.

Database searching resulted in the identification of 4795 citations. After removing duplicates, title and abstracts and full text were screened (figure 1). Additional articles were identified at the full text screening phase through reference screening of review of review articles and were subsequently screened for inclusion. A total of 80 reviews were included and informed the design of the evidence map. The PRISMA extension for Scoping Reviews (PRISMA-ScR) checklist is available as a supplementary document.

### Study characteristics

Of the 80 included reviews, publication years ranged from 2016 to 2020 with the majority of studies published in 2017 (N=18) and 2019 (N=19). The review types included systematic reviews without meta-analyses (N=51), systematic reviews with meta-analyses (N=15), meta-analyses without systematic review (N=9), and scoping reviews (N=5). Professional settings included healthcare and social assistance (N=38), education and training (N=2), and professional, scientific, and technical services (N=1). The remaining reviews were mixed or not stated (N=39). There were 17 intervention options and 12 outcomes reported across all reviews.

### Risk of bias

Sixty-six reviews scored higher than 50% of applicable AMSTAR 2 criteria and were assessed as having low-to-moderate risk of bias. Fourteen reviews fulfilled less than half of the criteria of the tool and thus were assessed as having a moderate-to-high risk of bias. The majority of reviews used systematic review methods and were of high quality for the most researched interventions (mindfulness/meditation, education/information provision and individual psychological therapies). Both high and low quality reviews are included in the evidence map.

### Evidence map

The evidence map comprises 17 intervention types that were designed to influence 12 outcomes [click here to access]. The interventions with the largest evidence base (ie, number of reviews of the intervention) were mindfulness/meditation (N=45 reviews), education and information provision (N=30 reviews) and individual psychological therapies (N=24 reviews). Interventions with the smallest evidence base were compassion fatigue, virtual reality, pharmacotherapy, stress management, and spiritual/religious interventions (N=1 review each). The most examined outcomes included burnout / stress reduction (N=65 reviews) and mental well-being (N=44 reviews), with the least examined being economic outcomes and absenteeism (N=1 review). It is these interventions and outcomes that will be the focus of the following results synthesis.

The direction of evidence is indicated using color and the size of the circles displays the relative number of reviews contributing to the evidence. The larger the circle, the more evidence there is for the interaction between the intervention and outcome. Links to the published journal articles for each circle are displayed on the right-hand side. Users can filter the rows and columns depending on their needs or interests. Intervention types with the largest evidence base are discussed in text below.

### Interventions

The most commonly reported interventions measured in reviews were mindfulness (N=42 reviews), education and information provision (N=29), and psychological therapies – individual (N=23). These interventions also had the greatest number of high quality reviews, which are presented below (the evidence map includes all interventions and outcomes, from both low and high quality reviews).

### Mindfulness

Mindfulness interventions had the greatest number (N=34) of reviews reporting high quality positive effects (table 1). Mindfulness is defined as an ‘intentional attentiveness’ to the present moment with openness, acceptance and curiosity (33). Reviews reporting mindfulness interventions examined the effects of these interventions on eleven outcomes (see supplementary material). The positive effect outcomes most reported for mindfulness interventions were for burnout / stress reduction (N=25/27 reviews) and mental well-being (N=15/16 reviews). There were also some reviews reporting ‘no conclusion’ for burnout / stress reduction, mental well-being, mental illness (N=2 each), and attitudinal or emotional measures (N=1). One review found neutral effects for mindfulness on productivity) and no reviews found negative effects.

**Table 1:** High quality reviews of mindfulness and mediation.

Author, year	Review type	Professional group	Direction of evidence	Outcome(s)	Quality* score	Additional interventions studied
Aryankhesal 2019 (49)	Systematic review	Health care and social assistance	Positive effect	Burnout / Stress	7/13	Organisational modifications; Education / information provision; Psychological therapies (individual); Mindfulness / meditation
Bartlett 2019 (50)	Systematic review and Meta-analysis	Mixed / not stated	Positive effect	Burnout / Stress	14/16	
			Positive effect	Fatigue		
			Positive effect	Mental Illness		
			Positive effect	Mental Well-being		
Brand 2017 (51)	Systematic review	Healthcare and social assistance	Positive effect	Mental Well-being	11/13	Mixed (Mindfulness / meditation; Education / information provision; Team-building activities; Organisational modifications)
Bresesti 2020 (52)	Systematic review	Healthcare and social assistance	Positive effect	Burnout / Stress	8/13	Mindfulness / meditation; Education / information provision; Organisational modifications
Brooks 2018 (46)	Systematic review	Mixed / not stated	No conclusion	Mental Illness	7/13	Psychological therapies (individual); Mindfulness / meditation
Burton 2017 (53)	Systematic review and Meta-analysis	Healthcare and social assistance	Positive effect	Burnout / Stress	9/16	
Busireddy 2017 (47)	Systematic review	Healthcare and social assistance	Positive effect	Burnout / Stress	11/16	Organisational modifications; Mindfulness / meditation
Cocker 2016 (54)	Systematic review	Healthcare and social assistance	No conclusion	Burnout / Stress	7/13	Mindfulness / meditation; Education / information provision; Arts-based interventions; Psychological therapies (group); Brain stimulation (psychological therapies (individual)
Emerson 2017 (55)	Systematic review	Education and training	No conclusion	Attitudinal and / or Emotional Measures	8/13	
			No conclusion	Burnout / Stress		
			No conclusion	Mental Illness		
			No conclusion	Mental Well-being		
			Positive effect	Attitudinal and / or Emotional Measures		
Dharmawardene 2016 (56)	Systematic review and Meta-analysis	Healthcare and social assistance	Positive effect	Attitudinal and / or Emotional Measures	10/16	
			Positive effect	Burnout / Stress		
			Positive effect	Mental Illness		
Dreison 2018 (57)	Meta-analysis	Healthcare and social assistance	Positive effect	Burnout / Stress	10/16	Education / information provision; Mindfulness / meditation; Psychological therapies (individual)
Ghawadra 2019 (58)	Systematic review	Healthcare and social assistance	Positive effect	Burnout / Stress	9/13	
			Positive effect	Employee Satisfaction		
			Positive effect	Mental Illness		
Gilmartin 2017 (59)	Systematic review	Healthcare and social assistance	Positive effect	Attitudinal and / or Emotional Measures	11/13	
			Positive effect	Burnout / Stress		
			Positive effect	Mental Illness		
			Positive effect	Mental Well-being		
			Neutral Effect	Productivity		
Haggman-Laitila 2018 (60)	Systematic review	Healthcare and social assistance	Positive effect	Burnout / Stress	8/13	Mindfulness / meditation; Spiritual / religious
			Positive effect	Mental Well-being		
Heckenberg 2018 (61)	Systematic review and Meta-analysis	Mixed / not stated	Positive effect	Burnout / Stress	11/16	
Howarth 2018 (45)	Systematic review	Mixed / not stated	Positive effect	Fatigue	10/13	
			Positive effect	Productivity		
Hwang 2017 (62)	Systematic review	Education and training	Positive effect	Burnout / Stress	9/13	
			Positive effect	Mental Well-being		
			Positive effect	Mental Illness		
			Positive effect	Productivity		
			Positive effect	Fatigue		
Ivandic 2017 (63)	Systematic review	Mixed / not stated	No Conclusion	Mental well-being	8/13	Mindfulness / meditation; Education / information provision
Janssen 2018 (64)	Systematic review	Mixed / not stated	Positive effect	Attitudinal and / or Emotional Measures	9/13	
			Positive effect	Burnout / Stress		
			Positive effect	Fatigue		
			Positive effect	Mental Illness		
			Positive effect	Mental Well-being		
Kunzler 2020 (...professionals) (65)	Systematic review	Healthcare and social assistance	Positive effect	Burnout / Stress	16/16	Psychological therapies (group), Mindfulness / meditation; Psychological therapies (individual)
			Positive effect	Mental Illness		
			Positive effect	Mental Well-being		
Kunzler 2020 (...students) (66)	Systematic review	Healthcare and social assistance	Positive effect	Burnout / Stress	16/16	Psychological therapies (group), Mindfulness / meditation; Psychological therapies (individual)
			Positive effect	Mental Illness		
			Positive effect	Mental Well-being		
Lee 2016 (67)	Meta-analysis	Healthcare and social assistance	Positive effect	Burnout / Stress	11/16	Psychological therapies (group); Education / information provision; Mindfulness / meditation; Peer support
Lomas 2019 (68)	Systematic review and Meta-analysis	Mixed / not stated	Positive effect	Attitudinal and / or Emotional Measures	11/16	
			Positive effect	Burnout / Stress		
			Positive effect	Mental Well-being		
			Positive effect	Productivity		
Luken 2016 (69)	Systematic review	Mixed / not stated	Positive effect	Burnout / Stress	8/13	
Murray 2016 (70)	Systematic review	Healthcare and social assistance	Positive effect	Mental Illness	11/13	Psychological therapies (individual); Education / Information provision; Mindfulness / meditation
			Positive effect	Mental Well-being		
Panagioti 2017 (71)	Meta-analysis	Healthcare and social assistance	Positive effect	Burnout / Stress	12/16	Mindfulness / meditation; Education / information provision; Exercise; Organisational modifications
Ryan 2017 (72)	Scoping review	Mixed / not stated	Positive effect	Burnout / Stress	7/13	
			Positive effect	Mental Well-being		
Soprovich 2020 (73)	Systematic review	Mixed / not stated	Positive effect	Fatigue	8/13	Exercise; Mindfulness / meditation; Education / Information provision
Stanulewicz 2019 (74)	Systematic review	Healthcare and social assistance	Positive effect	Burnout / Stress	11/13	Exercise; Mindfulness / meditation; Education / Information provision
			Positive effect	Mental Well-being		
			Positive effect	Physical Health		
			Positive effect	Productivity		
Suleiman-Martos 2020 (75)	Systematic review and meta-analysis	Healthcare and social assistance	Positive effect	Attitudinal and / or Emotional Measures	11/16	
			Positive effect	Burnout / Stress		
			Positive effect	Life Purpose and Satisfaction		
Trowbridge 2016 (76)	Systematic review	Healthcare and social assistance	Positive effect	Attitudinal and / or Emotional Measures	7/13	
			Positive effect	Productivity		
Vega-Escano 2020 (77)	Systematic review and Meta-analysis	Mixed / not stated	Positive effect	Fatigue	13/16	Psychological therapies (group); Mindfulness / meditation; Psychological therapies (individual)
Verbeek 2019 (78)	Scoping review	Mixed / not stated	Positive effect	Fatigue	7/13	Psychological therapies (group); Psychological therapies (individual)
West 2016 (79)	Meta-analysis	Healthcare and social assistance	Positive effect	Burnout / Stress	13/16	Organisational modifications; Education / information provision; Mindfulness / meditation
Wild 2020 (80)	Systematic review	Healthcare and social assistance	Positive effect	Mental Illness;	10/13	Mindfulness / meditation; Psychological therapies (individual); Psychological therapies (group); Exercise; Education / Information provision
			Positive effect	Mental Well-being		
			Positive effect	Physical Health		
Williams 2018 (81)	Systematic review	Healthcare and social assistance	Positive effect	Burnout / Stress	8/13	Team building; Exercise, Mindfulness / meditation; Psychological therapies (individual); Psychological therapies (group)
			Positive effect	Mental Illness		
			Positive effect	Mental Well-being		
			Positive effect	Physical Health		
Xu 2020 (82)	Systematic review	Healthcare and social assistance	Positive effect	Attitudinal and / or Emotional Measures	15/16	
			Positive effect	Burnout / Stress		
			Positive effect	Fatigue		
			Positive effect	Mental Illness		

* Quality of review refers to the quality assessed by two reviewers using AMSTAR 2.

### Education and information provision

Education and information provision interventions had the second greatest number of high quality reviews with positive effects (N=22; table 2). Education and information provision were defined as strategies to raise awareness of mental illness and how to manage it in workplaces. The outcomes with the largest evidence base for positive effects were burnout / stress reduction (N=9/12) and mental well-being (N=9/12). However, some reviews reported no conclusion or were neutral in their conclusions on these outcomes (N=3 for burnout / stress reduction, N=3 for mental well-being).No reviews found negative effects.

**Table 2:** High quality reviews of education and information provision.

Author, year	Review type	Professional group	Direction of evidence	Outcome(s)	Quality* score	Additional interventions studied
Aryankhesal 2019 (49)	Systematic review	Health care and social assistance	Positive effect	Burnout / Stress	7/13	Organisational modifications; Psychological therapies (individual); Mindfulness / meditation
Brand 2017 (51)	Systematic review	Health care and social assistance	Positive effect	Mental Well-being	11/13	Mixed (Mindfulness / meditation; Team-building activities; Organisational modifications)
Breseti 2020 (52)	Systematic review	Health care and social assistance	Positive effect	Burnout / Stress	8/13	Mindfulness / meditation; Organisational modifications
Brooks 2018 (46)	Systematic review	Mixed / not stated	Positive effect	Attitudinal and / or Emotional Measures	7/13	Psychological therapies (individual); Mindfulness / meditation
Busireddy 2017 (47)	Systematic review	Health care and social assistance	Positive effect	Attitudinal and / or Emotional Measures	11/16	Organisational modifications; Mindfulness / meditation
Cocker 2016 (54)	Systematic review	Healthcare and social assistance	No conclusions	Burnout / Stress	7/13	Mindfulness / meditation; Arts-based interventions; Psychological therapies (group); Brain stimulation (psychological therapies (individual)
Daniels 2017 (83)	Systematic review	Mixed / not stated	Positive effect	Mental Well-Being	9/13	
Dreison 2018 (57)	Meta-analysis	Health care and social assistance	Positive effect	Burnout / Stress	10/16	Mindfulness / meditation; Psychological therapies (individual)
Gayed 2018 (84)	Systematic review and meta-analysis	Mixed / not stated	Positive effect	Attitudinal and / or Emotional Measures	11/16	
			Neutral effect	Mental well-Being		
Haggman-Laitila 2018 (60)	Systematic review	Health care and social assistance	Positive effect	Fatigue	8/13	Mindfulness / meditation; Spiritual / religious;
			Positive effect	Mental Illness		
			Positive effect	Attitudinal and / or Emotional Measures		
			Positive effect	Burnout / Stress		
Hill 2016 (85)	Systematic review	Health care and social assistance	No conclusions	Burnout / Stress	9/13	Psychological therapies (individual); Psychological therapies (group)
			No conclusions	Mental Illness		
			No conclusions	Mental Well-Being		
			No conclusions	Fatigue		
Howarth 2018 (45)	Systematic review	Mixed / not stated	Positive effect	Mental Well-Being	10/13	Psychological therapies (individual); Mindfulness / meditation
			Positive effect	Mental Illness		
			Positive effect	Attitudinal and / or Emotional Measures		
			Positive effect	Productivity		
			Positive effect	Employee Satisfaction		
Ivandic 2017 (63)	Systematic review	Mixed / not stated	No conclusions	Mental Well-Being	8/13	Mindfulness / meditation
Kuster 2017 (86)	Systematic review	Mixed / not stated	No conclusions	Burnout / Stress	12/13	
Lee 2016 (67)	Meta-analysis	Healthcare and social assistance	Positive effect	Burnout / Stress	11/16	Psychological therapes (group); Mindfulness / meditation; Peer support
Murray 2016 (70)	Systematic review	Healthcare and social assistance	Positive effect	Mental Illness	11/13	Psychological therapies (individual); Mindfulness / meditation
			Positive effect	Mental Well-Being		
Nigatu 2019 (87)	Systematic review and Meta-analysis	Mixed / not stated	Positive effect	Mental Illness	11/16	Psychological therapies (individual); Psychological therapies (group); Exercise
Panagioti 2017 (71)	Meta-analysis	Healthcare and social assistance	Positive effect	Burnout / Stress	12/16	Mindfulness / meditation; Exercise; Organisational modifications
Soprovich 2020 (73)	Systematic review	Mixed / not stated	Positive effect	Fatigue	8/13	Exercise; Mindfulness / meditation
Stanulewicz 2019 (74)	Systematic review	Healthcare and social assistance	Positive effect	Mental Well-Being	11/13	Exercise; Mindfulness / meditation
			Positive effect	Burnout / Stress		
			Positive effect	Productivity		
			Positive effect	Physical Health		
Stuber 2020 (88)	Systematic review	Healthcare and social assistance	Positive effect	Mental Well-Being	9/13	
			Positive effect	Mental Illness		
Webster 2020 (89)	Scoping Review	Healthcare and social assistance	Positive effect	Mental Well-Being	7/13	Peer support; team building activities
West 2016 (79)	Meta-analysis	Healthcare and social assistance	Positive effect	Burnout / Stress	13/16	Organisational modifications; Mindfulness / meditation
Wild 2020 (80)	Systematic review	Healthcare and social assistance	Positive effect	Mental Illness	10/13	Mindfulness / meditation; Psychological therapies (individual); Psychological therapies (group); Exercise
			Positive effect	Mental Well-Being		
			Positive effect	Physical Health		
Xu 2020 (82)	Systematic review	Healthcare and social assistance	Positive effect	Burnout / Stress	15/16	Mindfulness / meditation; Psychological therapies (individual); Psychological therapies (group); Organisational modifications; Physical environmental modifications; Pharmacotherapies
			Positive effect	Attitudinal and / or Emotional Measures		
			Positive effect	Fatigue		
			Positive effect	Mental Illness		

*Quality of review refers to the quality assessed by two reviewers using AMSTAR 2.

### Psychological therapies – individual

Psychological therapies were categorized as either group-based or individual (for examples of group-based please see the evidence map [click here to access]. Examples of individual psychological therapies include cognitive behavioral therapy (CBT) or other therapies not involving pharmacotherapies, including access to employment assistance programs. There were 22 high quality reviews with 20 reporting positive outcomes for psychological therapies (table 3). The majority reported positive outcomes for burnout / stress reduction (N=11/13), mental illness (N=10/12), and mental well-being (N=8/9). Review authors also reported no conclusions for these outcomes, burnout / stress reduction (N=2), mental well-being (N=2), and mental illness (N=1). No reviews found negative effects.

**Table 3:** High quality reviews of psychological therapies.

Author, year	Review type	Professional group	Direction of Evidence	Outcome(s)	Quality* score	Additional interventions studied
Aryankhesal 2019 (49)	Systematic review	Health care and social assistance	Positive effect	Burnout / Stress	7/13	Organisational modifications; Education / information provision; Mindfulness / meditation; Team-building activities; Peer support; Mindfulness / meditation
Bellon 2019 (90)	Systematic review and meta-analysis	Mixed / not stated	Positive effect	Mental Illness	14/16	
Brooks 2018 (46)	Systematic review	Mixed / not stated	No conclusions	Mental Illness	7/13	Education / information provision; Mindfulness / meditation
Carolan 2017 (91)	Systematic review and Mmeta-analysis	Mixed / not stated	Positive effect	Mental Well-Being	12/16	
			Positive effect	Productivity		
Cocker 2016 (54)	Systematic review	Healthcare and social assistance	No conclusions	Burnout / Stress	7/13	Mindfulness / meditation; Arts-based interventions; Brain stimulation (psychological therapies (individual); Education / Information provision
Dreison 2018 (57)	Meta-analysis	Health care and social assistance	Positive effect	Burnout / Stress	10/16	Education / information provision; Mindfulness / meditation
Hill 2016 (85)	Systematic review	Health care and social assistance	No conclusions	Burnout / Stress	9/13	Education / information provision
			No conclusions	Mental Illness		
			No conclusions	Mental Well-Being		
			No conclusions	Fatigue		
Howarth 2018 (45)	Systematic review	Mixed / not stated	Positive effect	Fatigue	10/13	Education / information provision; Mindfulness / meditation
Kunzler 2020 (...professionals) (65)	Systematic review	Healthcare and social assistance	Positive effect	Mental Well-Being	16/16	Mindfulness / meditation
			Positive effect	Mental Illness		
			Positive effect	Burnout / Stress		
Kunzler 2020 (...students) (66)	Systematic review	Healthcare and social assistance	Positive effect	Mental Well-Being	16/16	Mindfulness / meditation
			Positive effect	Mental Illness		
			Positive effect	Burnout / Stress		
Maricutoiu 2016 (92)	Meta-analysis	Mixed / not stated	Positive effect	Burnout / Stress	10/16	Mixed
Murray 2016 (70)	Systematic review	Healthcare and social assistance	Positive effect	Mental Illness	10/16	Education / Information provision; Mindfulness / meditation
			Positive effect	Mental Well-Being		
Nigatu 2019 (87)	Meta-analysis	Mixed / not stated	Positive effect	Mental Illness	11/16	Exercise; Education / Information provision
Reeve 2018 (93)	Systematic review and meta-analysis	Healthcare and social assistance	Positive effect	Burnout / Stress	10/16	
			Positive effect	Mental Illness		
Romppanen 2017 (94)	Systematic review	Healthcare and social assistance	Positive effect	Mental Well-Being	8/13	
			Positive effect	Burnout / Stress		
Ryan 2017 (72)	Scoping review	Mixed / Not stated	Positive effect	Mental Well-Being	7/13	Mindfulness / meditation
			Positive effect	Burnout / Stress		
Slemp 2019 (95)	Meta-analysis	Mixed / not stated	Positive effect	Mental Illness	9/16	
			Positive effect	Burnout / Stress		
Vega-Escano 2020 (77)	Systematic review and meta-analysis	Mixed / not stated	Positive effect	Fatigue	13/16	Mindfulness / meditation;
Verbeek 2019 (78)	Scoping review	Mixed / not stated	Positive effect	Implementation	7/13	Mindfulness / meditation; Exercise; Organisational modifications
Wild 2020 (80)	Systematic review	Healthcare and social assistance	Positive effect	Mental Illness	10/13	Mindfulness / meditation; Exercise; Education / Information provision
			Positive effect	Mental Well-Being		
			Positive effect	Physical Health		
Williams 2018 (81)	Systematic review	Healthcare and social assistance	Positive effect	Mental Illness	8/13	Team building; Exercise, Mindfulness / meditation
			Positive effect	Mental Well-Being		
			Positive effect	Physical Health		
			Positive effect	Burnout / Stress		
Xu 2020 (82)	Systematic review	Healthcare and social assistance	Positive effect	Burnout / Stress	15/16	Mindfulness / meditation; Organisational modifications; Physical environmental modifications; Pharmacotherapies; Education / Information provision
			Positive effect	Attitudinal and / or Emotional Measures		
			Positive effect	Fatigue		
			Positive effect	Mental Illness		

*Quality of review refers to the quality assessed by two reviewers using AMSTAR 2.

### Outcomes

The most commonly reported outcomes measured in reviews were burnout / stress reduction (N=65 reviews) and mental well-being (N=44 reviews). Burnout / stress reduction was commonly measured using the Maslach Burnout Inventory (MBI) (34), Professional Quality of Life Scale (ProQOL) questionnaire (35), and Staff Stressor Questionnaire (SSQ) (36). On the other hand, the reporting of well-being was considerably heterogenous. Well-being is often used interchangeably with other terminology and there is no international consensus on its definition (2–5). Some reviews report studies that measure well-being using validated survey instruments such as the WHO-Five Wellbeing Index (37), SF-12 (38), the World Health Organization Health and Work Performance Questionnaire (HPQ) (39). Others reported researcher created, non-validated measures for well-being.

## Discussion

The aim of this evidence review was to synthesize existing literature on workplace mental well-being interventions. An evidence map was produced to present the 80 reviews that exist on the topic, including the intervention types examined within the reviews and the effects those interventions had on various outcomes related to workplace mental well-being. The evidence map demonstrated that most interventions designed to improve workplace mental well-being involve mindfulness/meditation, education and information provision or individual psychological therapies. Furthermore, these interventions had the most positive effects on burnout / stress reduction and mental well-being. Although the publication year for the inclusion of reviews was from 2016 onwards, the reviews themselves covered much longer time periods. Therefore, the scoping review represented a comprehensive coverage of existing literature in this field. Although the inclusion of reviews published prior to 2016 may have added to the review and evidence map, these reviews would not have encompassed more recent primary studies.

The variety of evidence presented in the map suggests there is a wide range of literature available on this topic. However previous reviews have expressed concern around the lack of available literature specific to intervention effectiveness (13, 14, 40, 41). Knowledge translation, where knowledge obtained by research and presented in the literature is translated for, and delivered to, the populations who use it, has often been an area that is not sufficiently supported, leading to the lack of implementation of research into practice (18).

Effective knowledge translation relies on gaining an understanding of the knowledge that exists, ideally via the identification of high-quality systematic reviews through scoping review methodology, and present the findings of these reviews in a simple-to-understand and apply way (eg, in an evidence map) (18). Importantly, the evidence must be presented in a practical and useful way to those who can make change. In the case of workplace mental well-being programs, this includes those who fund, design and implement the evidence-based interventions within workplaces. It is potentially here where the connection between research and practice has been lost in the area of workplace mental well-being programs, suggesting that new methods, such as evidence mapping, are needed to support the use of evidence-based interventions in the workplace.

Three intervention types that have been largely researched (mindfulness, education and information provision and individual psychological therapies) all had positive effects for burnout / stress reduction. This suggests that workplaces can choose these interventions to deliver in the workplace and hope for improved burnout / stress in the employees who participate. Having the freedom to choose from several interventions is another way to ensure the intervention fits within the workplace. However, although reducing burnout / stress might be an outcome of integrating one of the three interventions in the workplace, addressing the contributing organizational and system-level factors to burnout / stress (such as inappropriate workload, poor management support and low recognition) that are unique to the workplace is necessary to truly ensure a thriving workplace when it comes to mental well-being (17). In this case, it would be necessary for workplaces to generate specific interventions that address the contextual factors contributing to burnout / stress within their workplace (42).

There are numerous ways for workplaces to address mental health that stem from multiple theoretical backgrounds including psychology, public health, and medicine. LaMontagne and colleagues (43) argue that interventions to increase mental health in the workplace should prevent harm and promote the positive aspects of work (such as promoting positive leadership practices) in order to optimize prevention and effectively manage mental health. This leaves workplace managers and HR representatives a difficult task in assessing a large and complex literature about what does and does not work for their specific needs; they need reliable, clear and interpretable evidence to guide their work.

The use of both scoping review and evidence mapping methods in this study was a strength as the methods provide workplace and policy decision-makers with a large amount of evidence, interprets it for them and provides practical insights to assist them in implementing evidence-based interventions (22). Scoping reviews have a wider breadth than a traditional systematic review focusing on one intervention, as they are designed to examine the extent, range and nature of research activity and identify research gaps (20). We extended scoping review methods by undertaking quality appraisal of the included reviews and highlighting the areas in which there was high-quality review-level research evidence. This allowed us to make some conclusions about specific interventions and outcomes as well as paint a broad picture of research activity. In order to support the translation of theory into practice and policy, stakeholders need to be able to easily access high quality evidence (18, 44). This review aids this translation by providing an evidence map presenting an overview of the review level evidence. Additionally, it highlights the evidence gaps in the literature, supporting researchers who actively seek to improve the evidence in this area (22).

A weakness of this study lies in the inclusion of 80 reviews. Although identifying many relevant reviews is a strength, the heterogeneity of the reviews' aims and interventions reduces the ability to confidently conclude on the most effective workplace mental well-being interventions for particular outcomes. This is could be due to the broad inclusion criteria that focuses on both interventions and outcomes of review level evidence. In addition, well-being does not have a globally-recognized definition, and continues to be used interchangeably across primary and review level evidence (2–5). This additional heterogeneity makes it difficult to measure the overall effectiveness of interventions at the level of a scoping review. For this reason, we propose that the review and evidence map be considered a first step for employers, clinicians and researchers in identifying areas of relatively high and low research evidence. We do not propose that workplaces initiate interventions solely on the basis of the scoping review and evidence map; rather, the map is designed to reduce the risk of investing in areas in which there is little or no review-level evidence, and encourage consideration of interventions that have a stronger evidence base. Having identified evidence-based intervention areas using the map, the next step should be consultation with expert clinicians and practitioners to identify specific workplace needs, tailor interventions appropriately and evaluate outcomes. Ideally, this should be undertaken in collaboration with researchers in order to continue to build the evidence base.

A further gap in the identified literature was the examination of economic outcomes. Given the focus on the workplace setting, there is a clear need to evaluate the effectiveness of interventions on workplace productivity and other economic measures. Reducing the impact of mental illness on individuals should be the primary aim of all mental wellbeing interventions; however, economic data is also critical for leveraging investment in mental wellbeing from both industry and government.

Likewise, the intervention components, dose and delivery varied across reviews. For example, education and information were provided using digital and non-digital mediums. Digital channels included websites containing health information, SMS and smart phone applications (45). Non-digital interventions were delivered face-to-face via workshops and training or through paper-based mediums (46, 47). While for mindfulness, dosage varied across interventions from 10-minute self-guided practice to 25-minute daily practice face-to-face over eight weeks (48). However, this is an accepted drawback of the evidence map method, where the purpose of the map is to accurately outline the current status of evidence regardless of how it is presented (22). Future research should look to fill the evidence gap for the return on investment of workplace mental well-being interventions, specifically high quality economic evaluations. Return on investment is a crucial factor to convince businesses of the value of mental well-being interventions.

### Concluding remarks

The evidence-base for workplace mental well-being programs is broad in both the intervention types tested and the outcomes examined. Eighty reviews that mostly covered interventions related to mindfulness, education and information provision and individual psychological therapies were identified. The reviews examined the effects of these interventions on several outcomes, mostly burnout / stress reduction and mental illness and mental well-being. Research suggests there is a gap between knowledge and practice, that is, research takes many years to be implemented in practice. This gap presents several challenges to implementing mental well-being interventions in the workplace, including understanding which intervention has the highest quality evidence underpinning it, and deciding which will present the best return on investment. Finally, while improving the translation of tested interventions into practice, all interventions must be resilient enough to maintain effectiveness while having individual components changed to suit the targeted workplace.

## Supplementary material

Supplementary material
