# Supplementary material for: How effective are interventions in optimizing workplace mental health and well-being? A scoping review of reviews and evidence map
Source: Scand J Work Environ Health. 2023 May 1;49(4):235–48. doi: 10.5271/sjweh.4087 (PMC10713995; doi:10.5271/sjweh.4087)
Supplement: Supplementary material [file SJWEH-49-235-S001.pdf]

How effective are interventions in optimizing workplace mental health and well-being? A scoping review of reviews and evidence map1

by Alex Waddell, PhD,[2] Breanne Kunstler, PhD, Alyse Lennox, BPsych(Hons), Loyal Pattuwage, MPH, Emily AC Grundy, BPsychSc(Hons), Diki Tsering, MES, Patrick Olivier, PhD, Peter Bragge, PhD

1. Correspondence to: Alex Waddell, Department of Human-Centred Computing, Faculty of Information Technology, Monash University, Clayton Campus, Melbourne, VIC, 3800, Australia. [E-mail: alex.waddell@monash.edu]
2. Supplementary Materials

Example Search Strategy

Ovid Medline and Epub Ahead of Print, In-Process & Other Non-Indexed Citations, Daily and Versions(R) 1946 to October 09, 2020

#	Search string
1	(worker* or employee* or staff or labourer* or artisan or workforce or workmen or workman or workwoman or workwomen or workplace or work place or work-place or occupational setting* or work-related).ti,ab.
2	(job or work or vocation* or hired).ti,ab.
3	exp Personnel/
4	2 and 3
5	1 or 4
6	(stress or burnout or anxiety or depression or occupational health or occupational therap* or mental health or mental disorder* or mental illness* or psychological or quality of life or well-being or well-being or quality of working life or personal satisfaction or work satisfaction or life satisfaction or work functioning or work* standard* or happiness).ti,ab.
7	(work-stress or job-stress).ti,ab.
8	(Work Condition* or work-related or "work related").ti,ab.
9	6 or 7 or 8

10	(interven* or program* or activit* or participat* or counsell* or assistance or improve* or uplift or manage* or control or address* or promot*).ti,ab. or *Occupational Health/ or *Workplace/ or *Occupational Health Services/
11	9 and 10
12	(review or overview or synopsis or literature review or concept synthesis or conceptual framework synthesis model or conceptual review or critical interpretive synthesis or critical literature review or evidence synthesis or integrative review or integrative literature review or interpretive synthesis or knowledge synthesis or meta-aggregation or meta aggregation or meta-analysis or meta analysis or meta-ethnography or meta ethnography or meta-interpretation or meta interpretation or meta-interpretive or meta interpretive or meta-narrative or meta narrative or meta-review or meta review or meta-narrative or meta narrative or meta study or meta-synthesis or meta synthesis or mixed-methods review or mixed methods review or mixed-methods synthesis or mixed methods synthesis or mixed-methods systematic review or mixed methods systematic review or mixed studies review or mixed-studies review or narrative review or narrative synthesis or rapid review or realist review or realist synthesis or research synthesis or review of qualitative studies or scoping review or systematic literature review or systematic review or systematic synthesis or thematic review or thematic synthesis or qualitative meta-synthesis or qualitative meta synthesis or qualitative review or qualitative synthesis or horizon scan or Evidence map).ti,ab. (review or overview or synopsis or literature review or concept synthesis or conceptual framework synthesis model or conceptual review or critical interpretive synthesis or critical literature review or evidence synthesis or integrative review or integrative literature review or interpretive synthesis or knowledge synthesis or meta-aggregation or meta aggregation or meta-analysis or meta analysis or meta-ethnography or meta ethnography or meta-interpretation or meta interpretation or meta-interpretive or meta interpretive or meta-narrative or meta narrative or meta-review or meta review or meta-narrative or meta narrative or meta study or meta-synthesis or meta synthesis or mixed-methods review or mixed methods review or mixed-methods synthesis or mixed methods synthesis or mixed-methods systematic review or mixed methods systematic review or mixed studies review or mixed-studies review or narrative review or narrative synthesis or rapid review or realist review or realist synthesis or research synthesis or review of qualitative studies or scoping review or systematic literature review or systematic review or systematic synthesis or thematic review or thematic synthesis or qualitative meta-synthesis or qualitative meta synthesis or qualitative review or qualitative synthesis or horizon scan or Evidence map).ti,ab.
13	evidence adj2 map).ti,ab.
14	12 or 13
15	5 and 11 and 14

16	limit 15 to (english language and yr="2016 -Current")

Inclusion and Exclusion Criteria

	Inclusion Criteria	Exclusion Criteria
Study Type	Systematic or narrative reviews (of reviews, quantitative or qualitative studies)<br>Scoping reviews / evidence maps	Primary research studies<br>Review protocols<br>Conference abstracts, book chapters<br>Theses / dissertations
Population	Employees of an organisation	Sole traders / independent contractors who work as individuals
Study design	Reviews of effectiveness ('what works') studies	Reviews where intervention effectiveness is not the primary focus
Study setting	Countries comparable to Australia i.e. developed nation health system, democratic country<br>Any population in a workplace setting (including offsite, but funded by a workplace)<br>Include interventions funded by a workplace, but which occur outside of the workplace (e.g. counselling)	Countries not comparable to Australia i.e. LMIC, non-democratic<br>Any population outside of a workplace setting<br>Any intervention not funded by a workplace
Intervention	Workplace mental health interventions to maintain mental well-being<br>'Workplace' is defined as supported by a workplace (either through direct funding or enabling staff time to be used), within the workplace or non-workplace setting (e.g. gym, park, home)<br>'Maintain mental well-being' is defined as interventions to either prevent mental illness or maintain mental well-being, including stress / burnout reduction and resilience-based interventions – including those relating to dealing with the impact of COVID-19 - but NOT management of mental illness<br>Highlight interventions using digital technology	Management of mental illness such as depression, anxiety, PTSD and other conditions<br>Workers' Compensation schemes<br>Well-being interventions not supported by workplaces<br>Return-to-Work interventions where people have been on leave for burnout, stress mental health or other reasons<br>Enterprise / HR arrangements e.g. sick leave, maternity leave entitlements<br>Employee Assistance Programs (EAPs)
Outcome	All reported outcomes with an emphasis on<br>Physical / mental health and / or well-being<br>Productivity<br>Staff satisfaction / turnover	No exclusions

Publication Status	English language<br>Peer-reviewed journal publications or review reports from relevant Government / other institutions<br>Published from 2016 onwards (unless from Google Scholar search)	

Preferred Reporting Items for Systematic reviews and Meta-Analyses extension for Scoping Reviews (PRISMA-ScR) Checklist

From: Tricco AC, Lillie E, Zarin W, O'Brien KK, Colquhoun H, Levac D, et al. PRISMA Extension for Scoping Reviews (PRISMAScR): Checklist and Explanation. Ann Intern Med. 2018;169:467–473. doi: 10.7326/M18-0850.

ITEM NUMBER	PRISMA-ScR CHECKLIST ITEM	PAGE NUMBER REPORTED
1	TITLE: Title	1
2	ABSTRACT: Structured summary	1
3	INTRODUCTION: Rationale	4, 5
4	INTRODUCTION: Objectives	5, 6
5	METHODS: Protocol and registration	5
6	METHODS: Eligibility criteria	5, Supplementary material
7	METHODS: Information sources	5
8	METHODS: Search	Supplementary material
9	METHODS: Selection of sources of evidence	5, 6
10	METHODS: Data charting process	6, 7
11	METHODS: Data items	6, 7
12	METHODS: Critical appraisal of individual sources of evidence	6
13	METHODS: Synthesis of results	6, 7
14	RESULTS: Selection of sources of evidence	8, figure 1
15	RESULTS: Characteristics of sources of evidence	Table 1-3
16	RESULTS: Critical appraisal within sources of evidence	8, Table 1-3, Figure 2 (evidence map)
17	RESULTS: Results of individual sources of evidence	Table 1-3
18	RESULTS: Synthesis of results	8, 9, 10, Figure 2 (evidence map
19	DISCUSSION: Summary of evidence	11, 12
20	DISCUSSION: Limitations	12, 13
21	DISCUSSION: Conclusions	13, 14
22	FUNDING: Funding	14

Included Review Articles

1. Abdin S, Welch RK, Byron-Daniel J, Meyrick J. The effectiveness of physical activity interventions in improving well-being across office-based workplace settings: a systematic review. Vol. 160, Public Health. Elsevier B.V.; 2018. p. 70–6.

2. Ahola K, Toppinen-Tanner S, Seppänen J. Interventions to alleviate burnout symptoms and to support return to work among employees with burnout: Systematic review and meta-analysis. Vol. 4, Burnout Research. Elsevier GmbH; 2017. p. 1–11.

3. Aryankhesal A, Mohammadibakhsh R, Hamidi Y, Alidoost S, Behzadifar M, Sohrabi R, et al. Interventions on reducing burnout is physicians and nurses: A systematic review. Med J Islam Repub Iran [Internet]. 2019;33(77). Available from: https://doi.org/1

4. Bartlett L, Martin A, Neil AL, Memish K, Otahal P, Kilpatrick M. A Systematic Review and Meta-Analysis of Workplace Mindfulness Training Randomized Controlled Trials. J Occup Health Psychol. 2019;24(1):108–26.

5. Bellón JÁ, Conejo-Cerón S, Cortés-Abela C, Pena-Andreu JM, García-Rodríguez A, Moreno-Peral P. Effectiveness of psychological and educational interventions for the prevention of depression in the workplace: A systematic review and meta-analysis. Vol. 45, Scandinavian Journal of Work, Environment and Health. Nordic Association of Occupational Safety and Health; 2019. p. 324–32.

6. Bischoff LL, Otto AK, Hold C, Wollesen B. The effect of physical activity interventions on occupational stress for health personnel: A systematic review. Vol. 97, International Journal of Nursing Studies. Elsevier Ltd; 2019. p. 94–104.

7. Bordado Sköld M, Bayattork M, Andersen LL, Schlünssen V. Psychosocial effects of workplace exercise – A systematic review. Scand J Work Environ Heal. 2019;45(6):533–45.

8. Brand SL, Coon JT, Fleming LE, Carroll L, Bethel A, Wyatt K. Whole-system approaches to improving the health and well-being of healthcare workers: A systematic review. Vol. 12, PLoS ONE. Public Library of Science; 2017.

9. Bresesti I, Folgori L, De Bartolo P. Interventions to reduce occupational stress and burn out within neonatal intensive care units: A systematic review. Vol. 77, Occupational and Environmental Medicine. BMJ Publishing Group; 2020. p. 515–9.

10. Brooks SK, Dunn R, Amlôt R, Greenberg N, Rubin GJ. Training and post-disaster interventions for the psychological impacts on disaster-exposed employees: a systematic review. J Ment Heal. 2018 Feb 15;1–25.

11. Burton A, Burgess C, Dean S, Koutsopoulou GZ, Hugh-Jones S. How Effective are Mindfulness-Based Interventions for Reducing Stress Among Healthcare Professionals? A Systematic Review and Meta-Analysis. Vol. 33, Stress and Health. John Wiley and Sons Ltd; 2017. p. 3–13.

12. Busireddy KR, Miller JA, Ellison K, Ren V, Qayyum R, Panda M. Efficacy of Interventions to Reduce Resident Physician Burnout: A Systematic Review. J Grad Med Educ. 2017 Jun 1;9(3):294–301.

13. Carolan S, Harris PR, Cavanagh K. Improving employee well-being and effectiveness: Systematic review and meta-analysis of web-based psychological interventions delivered in the workplace. J Med Internet Res. 2017;19(7).

14. Cocchiara RA, Dorelli B, Gholamalishahi S, Longo W, Musumeci E, Mannocci A, et al. Tai chi and workplace wellness for health care workers: A systematic review. Int J Environ Res Public Health. 2020 Jan 1;17(1).

15. Cocchiara RA, Peruzzo M, Mannocci A, Ottolenghi L, Villari P, Polimeni A, et al. The use of yoga to manage stress and burnout in healthcare workers: A systematic review. Vol. 8, Journal of Clinical Medicine. MDPI; 2019.

16. Cocker F, Joss N. Compassion fatigue among healthcare, emergency and community service workers: A systematic review. Int J Environ Res Public Health. 2016;13(6):1–18.

17. Daniels K, Gedikli C, Watson D, Semkina A, Vaughn O. Job design, employment practices and well-being: a systematic review of intervention studies. Vol. 60, Ergonomics. Taylor and Francis Ltd.; 2017. p. 1177–96.

18. DeChant PF, Acs A, Rhee KB, Boulanger TS, Snowdon JL, Tutty MA, et al. Effect of Organization-Directed Workplace Interventions on Physician Burnout: A Systematic Review. Mayo Clin Proc Innov Qual Outcomes. 2019 Dec;3(4):384–408.

19. Dharmawardene M, Givens J, Wachholtz A, Makowski S, Tjia J. A systematic review and metaanalysis of meditative interventions for informal caregivers and health professionals. Vol. 6, BMJ Supportive and Palliative Care. BMJ Publishing Group; 2016. p. 160–9.

20. Dobson KS, Szeto A, Knaak S. The Working Mind: A Meta-Analysis of a Workplace Mental Health and Stigma Reduction Program. Can J Psychiatry. 2019;64(1_suppl):39S-47S.

21. Dreison K, Luther L, Bonfils K, Sliter M. Job Burnout in Mental Health Providers: A Meta-Analysis of 35 Years of Intervention Research. J Occup Health Psychol. 2018;23(1):18–30.

22. Elder EG, Johnston A, Wallis M, Crilly J. Work-based strategies/interventions to ameliorate stressors and foster coping for clinical staff working in emergency departments: a scoping review of the literature. Vol. 23, Australasian Emergency Care. Elsevier Australia; 2020. p. 181–92.

23. Emerson LM, Leyland A, Hudson K, Rowse G, Hanley P, Hugh-Jones S. Teaching Mindfulness to Teachers: a Systematic Review and Narrative Synthesis. Mindfulness (N Y). 2017;8(5):1136–49.

24. Gayed A, Milligan-Saville JS, Nicholas J, Bryan BT, LaMontagne AD, Milner A, et al. Effectiveness of training workplace managers to understand and support the mental health needs of employees: A systematic review and meta-analysis. Vol. 75, Occupational and Environmental Medicine. BMJ Publishing Group; 2018. p. 462–70.

25. Ghawadra SF, Abdullah KL, Choo WY, Phang CK. Mindfulness-based stress reduction for psychological distress among nurses: A systematic review. Vol. 28, Journal of Clinical Nursing. Blackwell Publishing Ltd; 2019. p. 3747–58.

26. Gilmartin H, Goyal A, Hamati MC, Mann J, Saint S, Chopra V. Brief Mindfulness Practices for Healthcare Providers – A Systematic Literature Review. Vol. 130, American Journal of Medicine. Elsevier Inc.; 2017. p. 1219.e1-1219.e17.

27. Gritzka S, Macintyre TE, Dörfel D, Baker-Blanc JL, Calogiuri G. The effects of workplace nature-based interventions on the mental health and well-being of employees: A systematic review. Front Psychiatry. 2020;11.

28. Guillaumie L, Boiral O, Champagne J. A mixed-methods systematic review of the effects of mindfulness on nurses. Vol. 73, Journal of Advanced Nursing. Blackwell Publishing Ltd; 2017. p. 1017–34.

29. Häggman-Laitila A, Romppanen J. Outcomes of interventions for nurse leaders' well-being at work: A quantitative systematic review. Vol. 74, Journal of Advanced Nursing. Blackwell Publishing Ltd; 2018. p. 34–44.

30. Heckenberg RA, Eddy P, Kent S, Wright BJ. Do workplace-based mindfulness meditation programs improve physiological indices of stress? A systematic review and meta-analysis. Vol. 114, Journal of Psychosomatic Research. Elsevier Inc.; 2018. p. 62–71.

31. Hill RC, Dempster M, Donnelly M, McCorry NK. Improving the well-being of staff who work in palliative care settings: A systematic review of psychosocial interventions. Vol. 30, Palliative Medicine. SAGE Publications Ltd; 2016. p. 825–33.

32. Howarth A, Quesada J, Silva J, Judycki S, Mills PR. The impact of digital health interventions on health-related outcomes in the workplace: A systematic review. Digit Heal. 2018 Jan;4:205520761877086.

33. Hwang YS, Bartlett B, Greben M, Hand K. A systematic review of mindfulness interventions for in-service teachers: A tool to enhance teacher well-being and performance. Teach Teach Educ. 2017;64:26–42.

34. Ivandic I, Freeman A, Birner U, Nowak D, Sabariego C. A systematic review of brief mental health and well-being interventions in organizational settings. Vol. 43, Scandinavian Journal of Work, Environment and Health. Nordic Association of Occupational Safety and Health; 2017. p. 99–108.

35. Jacobs S, Johnson S, Hassell K. Managing workplace stress in community pharmacy organisations: lessons from a review of the wider stress management and prevention literature. Int J Ph [Internet]. 2018 [cited 2022 Jun 8];26:28–38. Available from: https://academic.oup.com/ijpp/article/26/1/28/6099667

36. Jamieson SD, Tuckey MR. Mindfulness interventions in the workplace: A critique of the current state of the literature. J Occup Health Psychol. 2017 Apr 1;22(2):180–93.

37. Janssen M, Heerkens Y, Kuijer W, Van Der Heijden B, Engels J. Effects of mindfulness-based stress reduction on employees' mental health: A systematic review. Vol. 13, PLoS ONE. Public Library of Science; 2018.

38. Jaworska-Burzyńska L, Kanaffa-Kilijańska U, Przysiężna E, Szczepańska-Gieracha J. The role of therapy in reducing the risk of job burnout - A systematic review of literature. Vol. 18, Archives of Psychiatry and Psychotherapy. Polish Psychiatric Association; 2016. p. 43–52.

39. Kunzler AM, Helmreich I, Chmitorz A, König J, Binder H, Wessa M, et al. Psychological interventions to foster resilience in healthcare professionals. Vol. 2020, Cochrane Database of Systematic Reviews. John Wiley and Sons Ltd; 2020.

40. Kunzler AM, Helmreich I, König J, Chmitorz A, Wessa M, Binder H, et al. Psychological interventions to foster resilience in healthcare students. Cochrane Database Syst Rev. 2020 Jul 20;2020(7).

41. Kuster AT, Dalsbø TK, Luong Thanh BY, Agarwal A, Durand-Moreau Q V., Kirkehei I. Computer-based versus in-person interventions for preventing and reducing stress in workers. Vol. 2017, Cochrane Database of Systematic Reviews. John Wiley and Sons Ltd; 2017.

42. Lee HF, Kuo CC, Chien TW, Wang YR. A Meta-Analysis of the Effects of Coping Strategies on Reducing Nurse Burnout. Appl Nurs Res. 2016;31:100–10.

43. Li H, Zhao M, Shi Y, Xing Z, Li Y, Wang S, et al. The effectiveness of aromatherapy and massage on stress management in nurses: A systematic review. Vol. 28, Journal of Clinical Nursing. Blackwell Publishing Ltd; 2019. p. 372–85.

44. Lomas T, Medina JC, Ivtzan I, Rupprecht S, Eiroa-Orosa FJ. The impact of mindfulness on the well-being and performance of educators: A systematic review of the empirical literature. Teach Teach Educ. 2017;61:132–41.

45. Lomas T, Medina JC, Ivtzan I, Rupprecht S, Eiroa-Orosa FJ. Mindfulness-based interventions in the workplace: An inclusive systematic review and meta-analysis of their impact upon well-being. J Posit Psychol. 2019 Sep 3;14(5):625–40.

46. Lomas T, Medina JC, Ivtzan I, Rupprecht S, Hart R, Eiroa-Orosa FJ. The impact of mindfulness on well-being and performance in the workplace: an inclusive systematic review of the empirical literature. Eur J Work Organ Psychol. 2017 Jul 4;26(4):492–513.

47. Luken M, Sammons A. Systematic Review of Mindfulness Practice for Reducing Job Burnout. Am J Occup Ther. 2016;70(2):p1-7002250020.

48. Maricuţoiu LP, Sava FA, Butta O. The effectiveness of controlled interventions on employees' burnout: A meta-analysis. J Occup Organ Psychol. 2016 Mar 1;89(1):1–27.

49. Murray M, Murray L, Donnelly M. Systematic review of interventions to improve the psychological well-being of general practitioners. Vol. 17, BMC Family Practice. BioMed Central Ltd.; 2016.

50. Naylor M, Ridout B, Campbell A. A scoping review identifying the need for quality research on the use of virtual reality in workplace settings for stress management. Vol. 23, Cyberpsychology, Behavior, and Social Networking. Mary Ann Liebert Inc.; 2020. p. 506–18.

51. Nigatu YT, Huang J, Rao S, Gillis K, Merali Z, Wang JL. Indicated Prevention Interventions in the Workplace for Depressive Symptoms: A Systematic Review and Meta-analysis. Vol. 56, American Journal of Preventive Medicine. Elsevier Inc.; 2019. p. e23–33.

52. Ochentel O, Humphrey C, Pfeifer K. of Exercise Therapy in Persons with Burnout. A Systematic Review and Meta-Analysis [Internet]. Vol. 17, ©Journal of Sports Science and Medicine. 2018. Available from: http://www.jssm.org

53. Pachito D V., Eckeli AL, Desouky AS, Corbett MA, Partonen T, Rajaratnam SMW, et al. Workplace lighting for improving alertness and mood in daytime workers. Vol. 2018, Cochrane Database of Systematic Reviews. John Wiley and Sons Ltd; 2018.

54. Panagioti M, Panagopoulou E, Bower P, Lewith G, Kontopantelis E, Chew-Graham C, et al. Controlled interventions to reduce burnout in physicians a systematic review and meta-analysis. Vol. 177, JAMA Internal Medicine. American Medical Association; 2017. p. 195–205.

55. Park S, Jang MK. Associations Between Workplace Exercise Interventions and Job Stress Reduction: A Systematic Review. Work Heal Saf. 2019 Dec 1;67(12):592–601.

56. Phillips CS, Becker H. Systematic Review: Expressive arts interventions to address psychosocial stress in healthcare workers. Vol. 75, Journal of Advanced Nursing. Blackwell Publishing Ltd; 2019. p. 2285–98.

57. Puerto Valencia LM, Weber A, Spegel H, Bögle R, Selmani A, Heinze S, et al. Yoga in the workplace and health outcomes: A systematic review. Vol. 69, Occupational Medicine. Oxford University Press; 2019. p. 163–76.

58. Ravalier JM, Wegrzynek P, Lawton S. Systematic review: Complementary therapies and employee well-being. Occup Med (Chic Ill). 2016 Aug 1;66(6):428–36.

59. Reeve A, Tickle A, Moghaddam N. Are acceptance and commitment therapy-based interventions effective for reducing burnout in direct-care staff? A systematic review and meta-analysis. Vol. 23, Mental Health Review Journal. Emerald Group Holdings Ltd.; 2018. p. 131–55.

60. Richins MT, Gauntlett L, Tehrani N, Hesketh I, Weston D, Carter H, et al. Early Post-trauma Interventions in Organizations: A Scoping Review. Vol. 11, Frontiers in Psychology. Frontiers Media S.A.; 2020.

61. Rohlf VI. Interventions for occupational stress and compassion fatigue in animal care professionals-a systematic review. Traumatology (Tallahass Fla). 2018 Sep 1;24(3):186–92.

62. Romppanen J, Häggman-Laitila A. Interventions for nurses' well-being at work: a quantitative systematic review. Vol. 73, Journal of Advanced Nursing. Blackwell Publishing Ltd; 2017. p. 1555–69.

63. Ryan C, Bergin M, Chalder T, Wells JSG. Web-based interventions for the management of stress in the workplace: Focus, form, and efficacy. Vol. 59, Journal of Occupational Health. Japan Society for Occupational Health; 2017. p. 215–36.

64. Schoonover KL, Hall-Flavin D, Whitford K, Lussier M, Essary A, Lapid MI. Impact of Poetry on Empathy and Professional Burnout of Health-Care Workers: A Systematic Review. Vol. 35, Journal of Palliative Care. SAGE Publications Ltd; 2020. p. 127–32.

65. Slemp GR, Jach HK, Chia A, Loton D, Kern ML. Contemplative interventions and employee distress: A meta-analysis. Stress Heal. 2019;35:227–225.

66. Soprovich AL, Seaton CL, Bottorff JL, Duncan MJ, Caperchione CM, Oliffe JL, et al. A systematic review of workplace behavioral interventions to promote sleep health in men. Vol. 6, Sleep Health. Elsevier Inc; 2020. p. 418–30.

67. Stanulewicz N, Knox E, Narayanasamy M, Shivji N, Khunti K, Blake H. Effectiveness of lifestyle health promotion interventions for nurses: A systematic review. Vol. 17, International Journal of Environmental Research and Public Health. MDPI; 2020.

68. Stratton E, Lampit A, Choi I, Calvo RA, Harvey SB, Glozier N. Effectiveness of eHealth interventions for reducing mental health conditions in employees: A systematic review and meta-analysis. Vol. 12, PLoS ONE. Public Library of Science; 2017.

69. Suleiman-Martos N, Gomez-Urquiza JL, Aguayo-Estremera R, Cañadas-De La Fuente GA, De La Fuente-Solana EI, Albendín-García L. The effect of mindfulness training on burnout syndrome in nursing: A systematic review and meta-analysis. Vol. 76, Journal of Advanced Nursing. Blackwell Publishing Ltd; 2020. p. 1124–40.

70. Taylor C, Xyrichis A, Leamy MC, Reynolds E, Maben J. Can Schwartz Center Rounds support healthcare staff with emotional challenges at work, and how do they compare with other interventions aimed at providing similar support? A systematic review and scoping reviews. Vol. 8, BMJ Open. BMJ Publishing Group; 2018.

71. Trowbridge K, Mische Lawson L. Mindfulness-based interventions with social workers and the potential for enhanced patient-centered care: A systematic review of the literature. Vol. 55, Social Work in Health Care. Routledge; 2016. p. 101–24.

72. Vanhove AJ, Herian MN, Perez ALU, Harms PD, Lester PB. Can resilience be developed at work? A meta-analytic review of resilience-building programme effectiveness. J Occup Organ Psychol. 2016 Jun 1;89(2):278–307.

73. Vega-Escaño J, Porcel-Gálvez AM, de Diego-Cordero R, Romero-Sánchez JM, Romero-Saldaña M, Barrientos-Trigo S. Insomnia interventions in the workplace: A systematic review and meta-analysis. Int J Environ Res Public Health. 2020 Sep 1;17(17):1–18.

74. Verbeek J, Ruotsalainen J, Laitinen J, Korkiakangas E, Lusa S, Mänttäri S, et al. Interventions to enhance recovery in healthy workers; A scoping review. Vol. 69, Occupational Medicine. Oxford University Press; 2019. p. 54–63.

75. Watson D, Tregaskis O, Gedikli C, Vaughn O, Semkina A. Well-being through learning: a systematic review of learning interventions in the workplace and their impact on well-being. Vol. 27, European Journal of Work and Organizational Psychology. Routledge; 2018. p. 247–68.

76. Webster NL, Oyebode JR, Jenkins C, Bicknell S, Smythe A. Using technology to support the emotional and social well-being of nurses: A scoping review. Vol. 76, Journal of Advanced Nursing. Blackwell Publishing Ltd; 2020. p. 109–20.

77. West CP, Dyrbye LN, Erwin PJ, Shanafelt TD. Interventions to prevent and reduce physician burnout: a systematic review and meta-analysis. Lancet. 2016 Nov 5;388(10057):2272–81.

78. Wild J, El-Salahi S, Esposti MD. The Effectiveness of Interventions Aimed at Improving Well-Being and Resilience to Stress in First Responders: A Systematic Review. Vol. 25, European Psychologist. Hogrefe Publishing GmbH; 2020. p. 252–71.

79. Williams SP, Malik HT, Nicolay CR, Chaturvedi S, Darzi A, Purkayastha S. Interventions to improve employee health and well-being within health care organizations: A systematic review. Vol. 37, Journal of healthcare risk management : the journal of the American Society for Healthcare Risk Management. 2018. p. 25–51.

80. Xu H, Kynoch K, Tuckett A, Eley R. Effectiveness of interventions to reduce emergency department staff occupational stress and/or burnout: A systematic review. Vol. 18, JBI Evidence Synthesis. Lippincott Williams and Wilkins; 2020. p. 1156–88.
